# Case report: Difference in outcomes between two cases of Hailey-Hailey disease treated with apremilast

**DOI:** 10.3389/fgene.2022.884359

**Published:** 2022-09-30

**Authors:** Misako Yamaga, Toshinari Miyauchi, Jin Teng Peh, Sota Itamoto, Yosuke Mai, Hiroaki Iwata, Toshifumi Nomura, Hideyuki Ujiie

**Affiliations:** ^1^ Department of Dermatology, Faculty of Medicine and Graduate School of Medicine, Hokkaido University, Sapporo, Japan; ^2^ Department of Dermatology, Faculty of Medicine, University of Tsukuba, Tsukuba, Japan

**Keywords:** Hailey-Hailey disease, apremilast, familial benign chronic pemphigus, ATP2C1, phosphodiesterase-4 inhibitor

## Abstract

Hailey-Hailey disease (HHD) is a rare autosomal dominant acantholytic dermatosis clinically characterized by recurrent erythematous plaques and erosions mainly on the intertriginous regions. Although HHD seriously affects quality of life, conventional treatments often fail to provide long-term relief for most patients. The effectiveness of apremilast, a phosphodiesterase-4 inhibitor, against severe HHD was first reported in 2018, and after further testing, this agent is currently expected to be established as an efficacious and safe therapeutic option. Here we report two cases of HHD treated with apremilast which showed opposite outcomes. Although the case with extremely severe symptoms showed remarkable and long-lasting improvement with apremilast used after acute treatment with oral corticosteroid, the other case, with milder symptoms treated only with apremilast, showed no improvement. Our transcriptome analysis using skin samples collected prior to apremilast administration revealed the involvement of the NF-κB signaling pathway, which is related to the responses to bacteria and other organisms. However, this pathway was more strongly activated in case 2 than in case 1, suggesting that the steroid treatment preceding apremilast may have been effective and supportive in the apremilast-responding case. One of the two cases highlights the potential of apremilast as a treatment option for HHD, but the other underlines the difficulties in managing HHD and the complexity of the disease background. The accumulation of cases and larger clinical studies are expected to precisely evaluate the safety and efficacy of apremilast, and the potential for therapies in combination with conventional treatments.

## 1 Introduction

Hailey-Hailey disease (HHD), also known as familial benign chronic pemphigus, is characterized by recurrent erythematous plaques and erosions mainly on the intertriginous regions (Lagha et al., 2020). It has autosomal dominant inheritance and is caused by loss-of-function mutations in *ATP2C1* (Lagha et al., 2020). ATP2C1 maintains normal intracellular concentrations of Ca^2+^/Mn^2+^ via transporting Ca^2+^/Mn^2+^ into the Golgi apparatus. Its mutations are reportedly responsible for abnormal cytosolic Ca^2+^/Mn^2+^ levels, resulting in the clinical manifestations of HHD. Treatment options for HHD include topical corticosteroids, topical calcineurin inhibitors, oral antimicrobial agents, oral retinoids, Botulinum toxin type A, and YAG lasers, though all fail to provide long-term relief for most patients. Furthermore, environmental factors and genetic modifiers may also affect the clinical variability or severity of HHD, making the management of the symptoms challenging. In 2018, the effectiveness of apremilast, a phosphodiesterase-4 (PDE4) inhibitor, on severe HHD was first reported (Kieffer et al., 2018), and it is currently expected to be established as an efficacious and safe therapeutic option. Here we report two cases of HHD treated with apremilast, which showed opposite outcomes.

## 2 Materials and methods

### 2.1 Human subjects and study approval

Individuals participating in the study provided written informed consent, in compliance with the Declaration of Helsinki. This study was approved by the Institutional Review Board of the Hokkaido University Graduate School of Medicine (project No. 14-063). All individuals provided peripheral blood samples and/or whole skin samples.

### 2.2 Genomic DNA extraction and sanger sequencing

For mutation analysis, genomic DNA from the individuals’ peripheral blood was extracted using a QIAamp DNA Blood Maxi Kit (Qiagen).

Exons and exon-intron boundaries in *ATP2C1* (RefSeq accession number NM_014382.5) were amplified via PCR using AmpliTaq Gold PCR Master Mix (Thermo Fisher Scientific). Primer sequences and PCR conditions are available upon request. PCR amplicons were treated with ExoSAP-IT reagent (Affymetrix), and the sequencing reaction was performed using BigDye Terminator version 3.1 (Thermo Fisher Scientific). Sequence data were obtained using an ABI 3130xl genetic analyzer (Applied Biosystems).

### 2.3 RNA extraction and quantitative PCR

RNA extraction and reverse transcription were performed using a RNeasy Mini Kit (Qiagen) and ReverTra Ace qPCR RT Kit (TOYOBO). Quantitative real-time PCR was carried out using the StepOnePlus Real-Time PCR System (Thermo Fisher Scientific) with TaqMan Fast Advanced Master Mix (Thermo Fisher Scientific) and TaqMan MGB probes (Thermo Fisher Scientific), according to the manufacturer’s instructions. The TaqMan probes used in this analysis were as follows: *ATP2C1* (Hs00995930_m1), *IL8* (Hs00174103_m1), *CCL20* (Hs003554 76_m1), and *ACTB* (Hs01060665_g1). *ATP2C1* expression values were normalized to *ACTB* levels, and relative expression levels were calculated using the ΔΔCt method.

### 2.4 RNA sequencing and data analysis

The library preparation and sequencing were performed by RIKEN Genesis. Briefly, the library was prepared using the TruSeq Stranded mRNA Library Prep Kit according to the manufacturer’s instructions. The libraries were sequenced at multiplex Paired-End 100 bp on the Illumina NovaSeq 6000. Reads were mapped to hg38 with Gencode v.39 annotations using STAR (v.2.7.10a) (Dobin et al., 2013). Gene expression levels were quantified using RSEM (v.1.3.3) (Li and Dewey, 2011). Read counts were analyzed using integrated Differential Expression and Pathway analysis (iDEP) (Ge et al., 2018). Genes with low levels of expression (<0.5 counts per million in all samples) were removed from the analysis. Gene clusters identified by k-means were analyzed by enrichment analysis based on Gene Ontology (GO) (Ashburner et al., 2000) and TRRUST (Han et al., 2018). For the comparison among HHD patients, we selected a gene set using a fold-change threshold of >5. This gene set was analyzed by enrichment analysis based on GO and TRRUST using Metascape (Zhou et al., 2019).

## 3 Case description

### 3.1 Case 1

**FIGURE 1:**
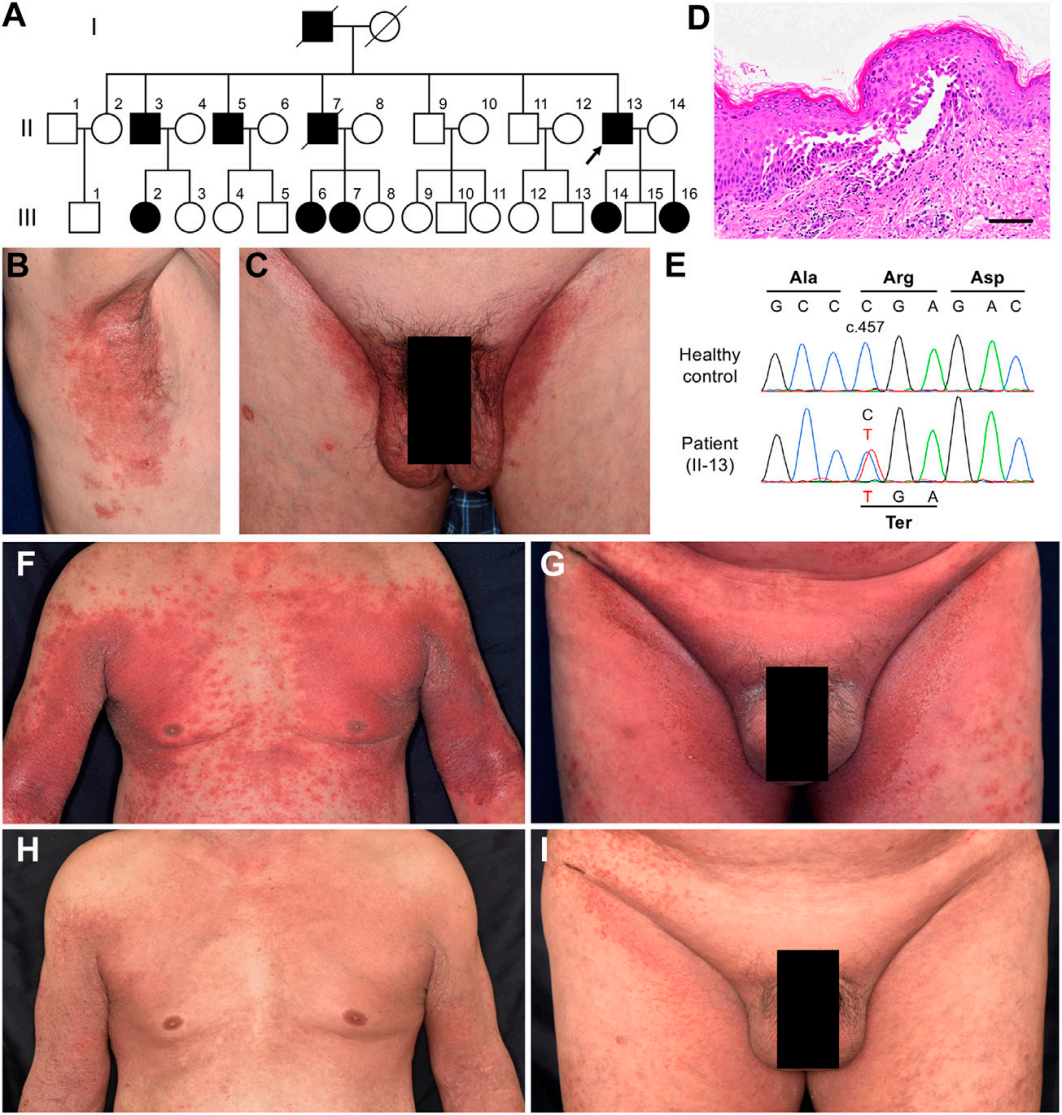
Clinical, histological, and genetic features of Case 1. **(A)** Pedigree of the family with HHD. Affected individuals are represented by a solid square or circle, depending upon the gender, and the proband (the present case) is indicated by an arrow. **(B, C)** Clinical findings at the first visit to our hospital. **(D)** Pathological features of the lesion on the right axilla. Hematoxylin and eosin staining (scale bars, 100 μm). **(E)** The patient is heterozygous for a nonsense mutation **(C)** c.457C > T (p. Arg153Ter) in *ATP2C1*. **(F, G)** The most severe state, at the third hospitalization. **(H, I)** Clinical improvement after a month of treatment with apremilast following the initial 2-week treatment with oral corticosteroid.

A 71-year-old man was referred to our hospital with lesions on the intertriginous areas presenting since his 40s. He had a family history of similar symptoms (Figure 1A) and had been histologically diagnosed with HHD. The effectiveness of treatments with topical corticosteroids and etretinate were very limited, and the symptoms showed exacerbation, especially in summer. Physical examination revealed well-circumscribed and painful erosions and erythema on the axillae and inguinal areas (Figures 1B,C). Our histological re-evaluation showed prominent spinous layer acantholysis (Figure 1D). Direct immunofluorescence was negative. Mutation analysis led to the identification of a heterozygous nonsense mutation c.457C>T (p. Arg153Ter) in *ATP2C1* and the HHD diagnosis was confirmed (Figure 1E).

He was repeatedly hospitalized, and treated with topical corticosteroid, increased amounts of etretinate, and/or antibiotics, and showed temporary improvement but no long-term efficacy. At the third hospitalization, the lesions had spread to around 50% of the body surface area (BSA) (Figures 1F,G). Use of 0.5 mg/kg/day of oral corticosteroid in acute management improved lesions by 20%. We then decided to initiate apremilast with the patient’s consent. He started taking 30 mg apremilast twice daily after completing a 6-days titration period, resulting in drastic improvement of the lesions within a month (Figures 1H,I). Notably, this efficacy has lasted for over 2.5 years with occasional minor flare-ups but no side effects.

### 3.2 Case 2

**FIGURE 2:**
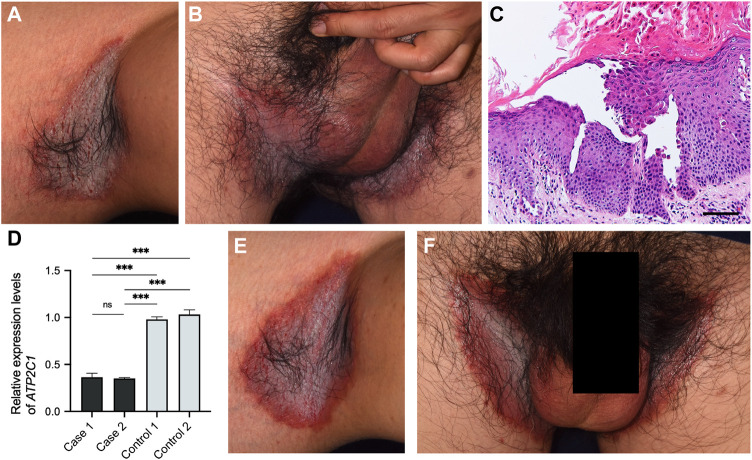
Clinical and histological features of Case 2. **(A, B)** Clinical findings at the first visit to our hospital. **(C)** Pathological features of the lesion on the right axilla. Hematoxylin and eosin staining (scale bars, 100 μm). **(D)** Gene expression levels of *ATP2C1* in whole skin samples taken from HHD patients and healthy controls. Statistical significance was calculated using one-way ANOVA with multiple comparisons test. Error bars represent SD. ***p < 0.001; ns, not significant. **(E, F)** No improvement after three months of treatment with apremilast.

A 39-year-old man presented to our hospital with lesions on the intertriginous regions that had continued to emerge/appear since he was 30. He had no family history of these symptoms. Clinically, he had painful erosions on the axillae and inguinal areas with strong odour (Figures 2A,B). Histologic findings from right axilla showed obvious acantholysis and hyperkeratosis (Figure 2C). Although no loss-of-function mutations were detected in *ATP2C1*, expression levels of this gene in the lesional skin decreased to levels similar to Case 1 compared to healthy controls, suggesting other factors affecting *ATP2C1* expression (Figure 2D). Considering the fact that *ATP2C1* mutations have not been identified in a few HHD cases (Deng and Xiao, 2017), this case was also considered to be consistent with HHD. The patient had been treated with etretinate and oral/topical corticosteroids, all of which had shown very limited effectiveness. Furthermore, although the BSA of the lesions was at most 8%, this had a serious impact on his daily life. We therefore initiated apremilast (60 mg/day) with the patient’s consent. However, as no improvement was observed after 3 months, we discontinued it. The patient is currently receiving symptomatic treatments with etretinate, oral clindamycin and adhesive dressings such as Mepilex Light (Mölnlycke Health Care AB).

## 4 Transcriptome analysis results

**FIGURE 3:**
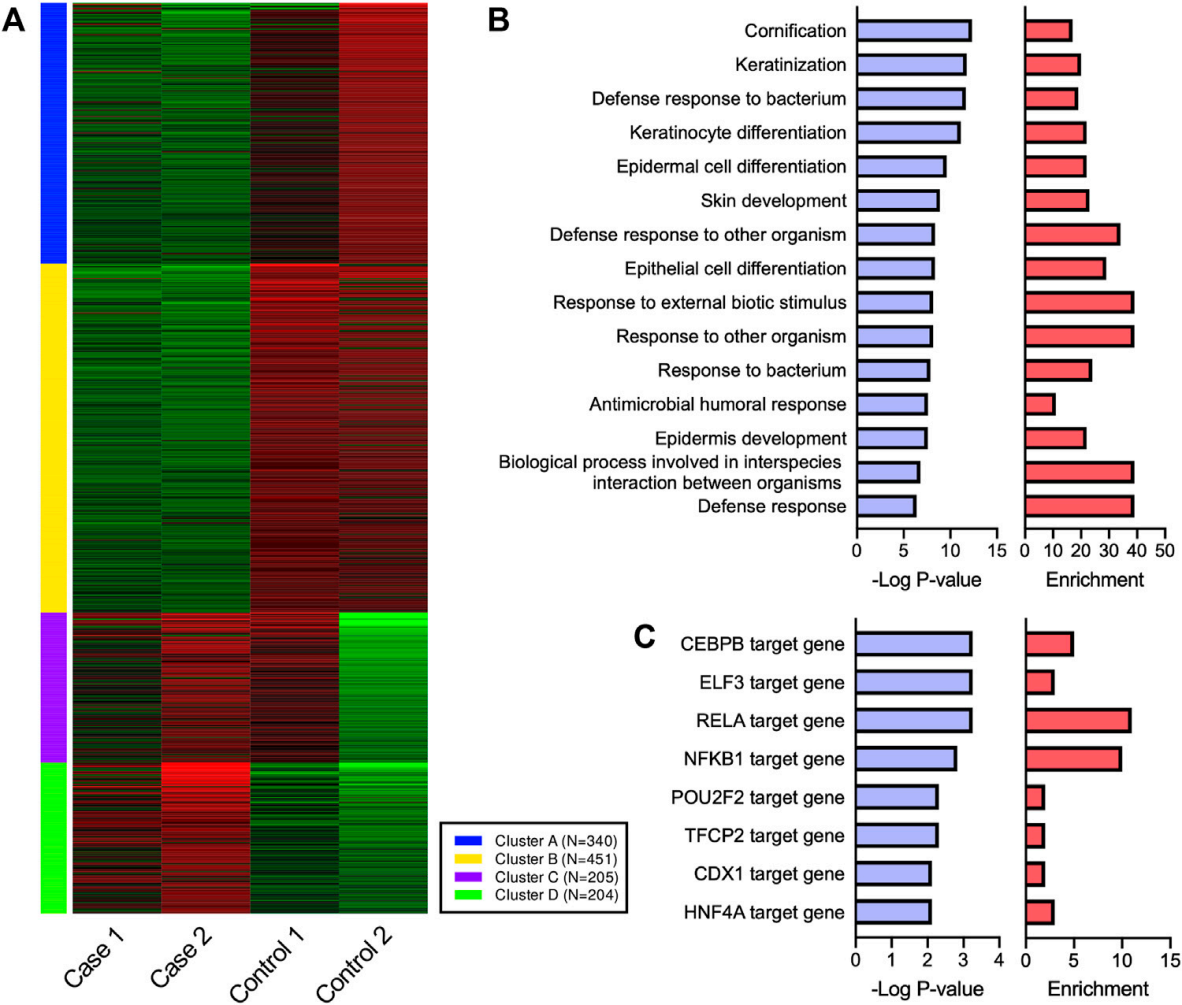
Transcriptional profiles of HHD and healthy control skin. **(A)** Four gene clusters identified by k-means based on the most variable 1,200 genes. **(B,C)** GO terms and TRRUST transcription factor-target interactions enriched in cluster D. RELA and NFKB1 are major components of NF-κB family.

To evaluate the inflammation status of HHD lesional skin, transcriptome analysis using RNA-seq was performed, with a comparison to two healthy control samples. Although the skin samples were collected before the initiation of apremilast in both cases, case 1 had been treated with oral corticosteroid prior to collecting the sample, which could be alter the inflammation states. The k-means clustering confirmed that the healthy control samples differed from the HHD samples in terms of transcriptional profiles (Figure 3A). Interestingly, GO terms enriched in cluster D showed defense responses to bacteria and other organisms as well as keratinization (Figure 3B, Supplementary Figure S1). In addition, TRRUST transcription factor-target interactions enriched in cluster D were partially related to the NF-κB signaling pathway (Figure 3C, Supplementary Figure S2).

**FIGURE 4:**
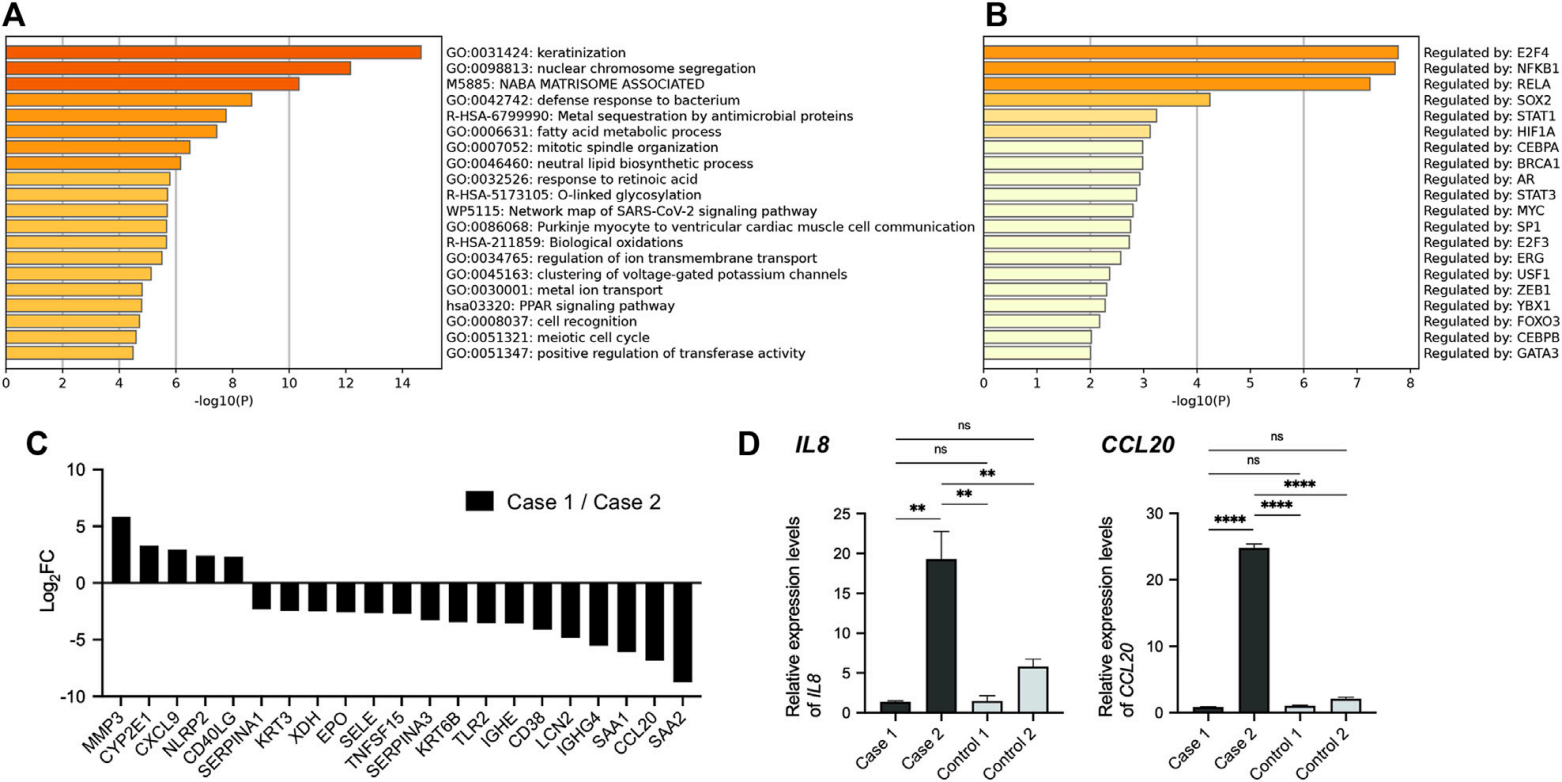
Comparison of transcriptional profiles among HHD patients. **(A)** GO terms related to the gene sets which had five-fold changes between HHD patients. **(B)** TRRUST analysis revealed the involvement of NFKB1 and RELA. **(C)** Expression profile of NF-κB target genes in HHD patients. Each bar represents the ratio of expression level in case 1 to that in case 2 for indicated gene. **(D)** Gene expression levels of *IL8* and *CCL20* in whole skin samples. Statistical significance was calculated using one-way ANOVA with multiple comparisons test. Error bars represent SD. **p < 0.01, ****p < 0.0001; ns, not significant.

We next compared an apremilast-responding patient (case 1) to an apremilast non-responding patient (case 2). We selected the gene set which had five-fold changes between these patients and performed an enrichment analysis (Figures 4A,B). Although few GO terms related to immune responses were enriched in this dataset (Figure 4A), TRRUST analysis suggested that the NF-κB pathway may have been involved in the differences between these patients (Figure 4B). We also compared the expression profile of NF-κB target genes in these patients, which suggesting that the NF-κB pathway might be upregulated in case 2 (Figure 4C). Indeed, gene expression levels of *IL8* and *CCL20*, which encode inflammatory chemokines commonly related to the NF-kB pathway in epidermis, were markedly upregulated in case 2. Conversely, these expression levels were suppressed to normal levels in case 1 (Figure 4D).

## 5 Discussion

Apremilast is an oral PDE4 inhibitor, which inhibits PDE4 and increases intracellular cAMP levels, suppressing the release of inflammatory cytokines (Zebda and Paller, 2018). It is widely recognized as a therapeutic option for psoriasis and psoriatic arthritis, and recently its effectiveness in the treatment of Behçet’s disease, hidradenitis suppurativa, sarcoidosis, and pemphigus vulgaris has been reported (Nassim et al., 2018; Meier et al., 2020.). It suggests its potential as a therapy for a variety of inflammatory skin diseases. Given its efficacy with these conditions, we can hypothesize that apremilast shows efficacy in suppressing secondary inflammations due to the destabilization of epidermal integrity in HHD. However, the functional mechanisms of apremilast on HHD and the involvement of inflammation in HHD remain unclear.

To our knowledge, there have been five reports about the efficacy of apremilast against HHD (Table 1) (Kieffer et al., 2018; Di Altobrando et al., 2020; Riquelme-Mc Loughlin et al., 2020; Siliquini et al., 2021; Yoto et al., 2021). In every case, apremilast was initiated after inadequate response to conventional treatments. Eight out of 15 cases showed good clinical courses with apremilast and its effectiveness was apparent within the first 2 months. Some cases had minor flare-ups during this period, which were described as manageable with topical treatments. However, one patient had to discontinue use due to subsequent flare-ups.

**TABLE 1:** Summary of all published work on HHD treated with apremilast.

No	Age/Sex	Outcome	Time taken for improvement	Follow-up period	Adverse effect	References
1	50s / F	Improved	4w	8m	-	Kieffer et al. (2018)
2	60s / F	Improved	4w	5m	Diarrhea	-
3	50s / F	Improved	4w	6m	-	-
4	50s / M	Improved, but administration discontinued due to new flare-ups	4w	10m	Myalgia, Diarrhea	-
5	68 / F	Improved	4w	12m	-	Di Altobrando et al. (2020)
6	58 / F	No Improvement	-	33w	Dyspepsia, GERD	Riquelme-Mc Loughlin et al. (2020)
7	54 / F	No Improvement	-	28w	-	-
8	50 / M	No Improvement	-	40w	-	-
9	36 / M	No Improvement	-	12w	-	-
10	55 / F	Stop administration	-	Intermittent	Diarrhea, Nausea	-
11	42 / F	Improved	8w	10m	Mild headache	Siliquini et al. (2020)
12	28 / M	Improved	2w	2y	-	Yoto et al. (2021)
13	35 / F	Improved	2w	1.5y	Headache	-
14	71 / M	Improved	4w	2.5y	-	Our cases
15	39 / M	No improvement	-	3m	-	-

Abbreviations: F, Female; M, Male; GERD, Gastroesophageal reflux disease.

In case 1, the widespread symptoms were extremely severe, but apremilast in conjunction with acute management with oral corticosteroid worked remarkably well. Furthermore, the patient had a satisfactory course over a follow-up period of more than 2.5 years, suggesting that long-lasting efficacy might be expected in patients whose lesions respond to this treatment. Furthermore, Yoto *et al.* reported two cases of HHD treated with apremilast with no recurrence for up to 2 years (Yoto et al., 2021). On the other hand, Riquelme-Mc Loughlin et al. (2020) used apremilast with five patients, but none of them showed any improvement. Apremilast was similarly ineffective in Case 2, even though it was less severe than Case 1.

As it was difficult to predict the effectiveness of apremilast based on clinical features, we used a transcriptome analytical approach, although the information was limited due to the small number of cases. A comparison between our two cases suggests that the NF-κB pathway was more strongly activated in case 2. Conversely, part of the gene expression related to this pathway was suppressed to normal levels in case 1. This may be largely due to the oral corticosteroid treatment in case 1 prior to apremilast initiation. Although extensive skin symptoms were still observed clinically, the acute treatment possibly suppressed most of the inflammation. In contrast, the defense responses to bacteria and other organisms were seen as shared pathways in both HHD samples. Moreover, NF-κB was involved in these pathways. It is easy to speculate the moist areas, such as intertriginous regions which are commonly affected in HHD, have distinct compositions of skin microbiome. The changes in the microbiome balance and the transition to infection may be important factors in the induction of inflammation and the relapse or exacerbation of HHD symptoms. In addition, a complex interplay of other factors, such as seasons, perspiration, and frictional stress, may result in cases where inflammation cannot be suppressed with apremilast alone. The potential for combination therapies, such as apremilast with antibacterial agents or oral steroids, should be explored in the future.

The present cases further highlight the potential of apremilast as a treatment option for HHD, especially in cases where conventional treatments have failed. However, they also highlight that there are, and may be, cases where this treatment is ineffective. As HHD seriously affects patients’ quality of life, larger clinical studies are warranted to precisely evaluate apremilast’s efficacy and safety.

## Data Availability

The datasets for this article are not publicly available due to concerns regarding participant/patient anonymity. Requests to access the datasets should be directed to the corresponding author.

## Supplementary material

Supplementary FIGURE S1 Enriched pathways for each cluster based on GO database.

Supplementary Figure S2 TRRUST analysis for each cluster.
