# Supplementary figures and images for: Case report: Difference in outcomes between two cases of Hailey-Hailey disease treated with apremilast
Source: Front Genet. 2022 Sep 30;13:884359. doi: 10.3389/fgene.2022.884359 (PMC9583697; doi:10.3389/fgene.2022.884359)

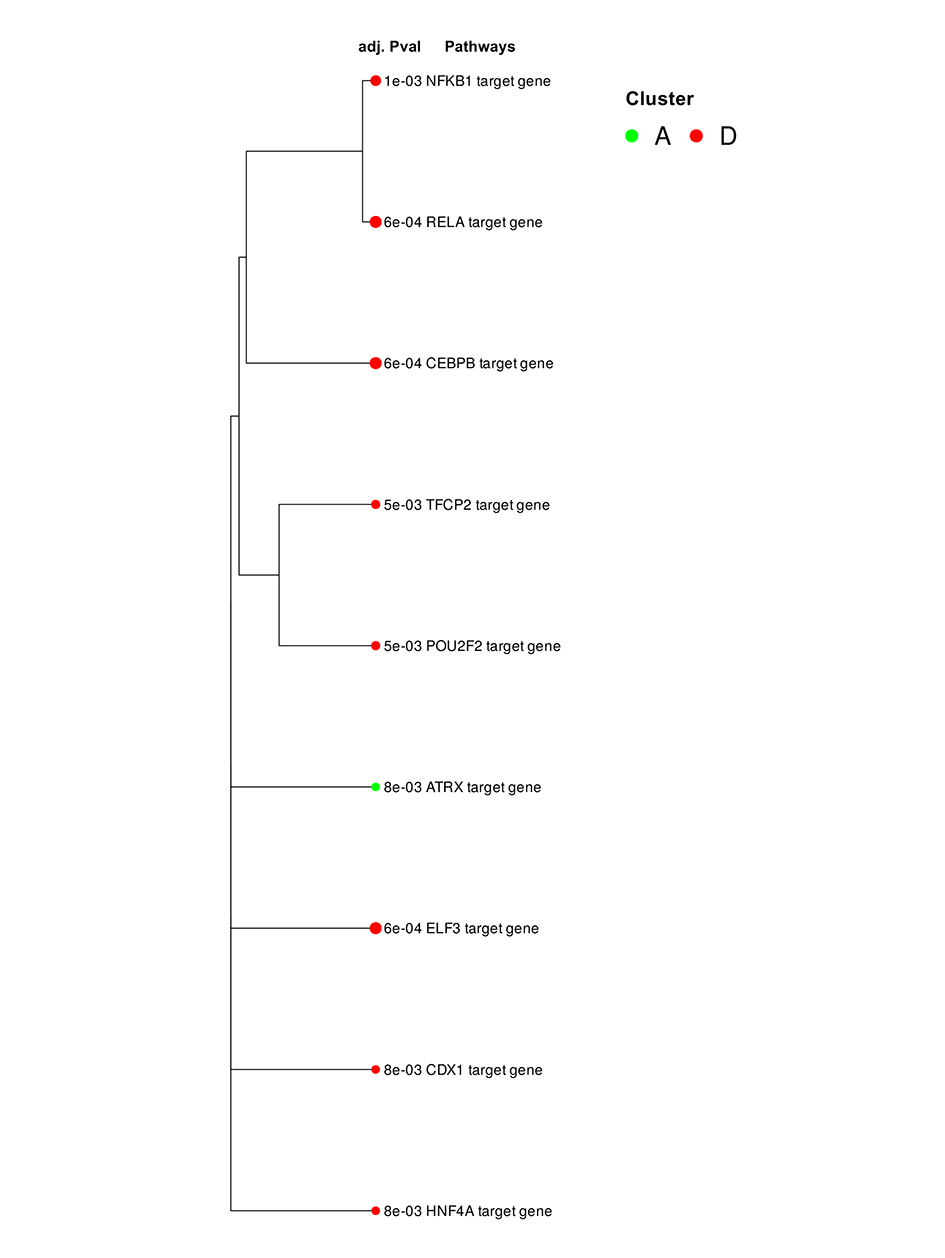

Supplement: Supplementary file 1 [file Image2.tif]

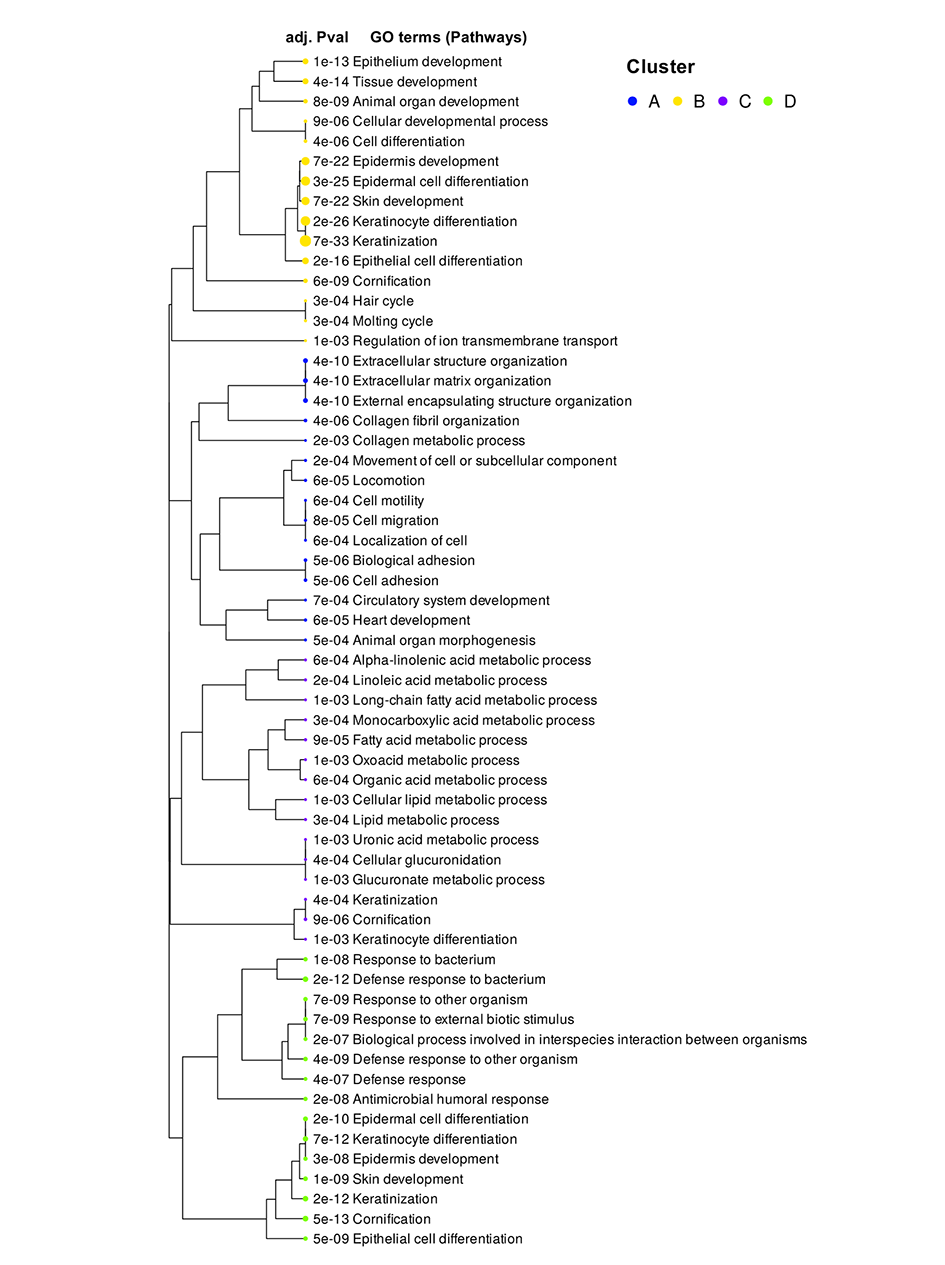

Supplement: Supplementary file 2 [file Image1.tif]
